# *Plau* and *Tgfbr3* are YAP-regulated genes that promote keratinocyte proliferation

**DOI:** 10.1038/s41419-018-1141-5

**Published:** 2018-10-31

**Authors:** Susan M. Corley, Veronica Mendoza-Reinoso, Nichole Giles, Emma Suwanun Singer, John E. Common, Marc R. Wilkins, Annemiek Beverdam

**Affiliations:** 1 Systems Biology Initiative, School of Biotechnology and Biomolecular Sciences, UNSW Australia, Sydney, New South Wales Australia; 2 School of Medical Sciences, UNSW Australia, Sydney, New South Wales Australia; 3 Institute of Medical Biology, A*STAR, 8A Biomedical Grove, Immunos, Singapore, 138648 Singapore; 4 Skin Research Institute of Singapore, A*STAR, 8A Biomedical Grove, Immunos, Singapore, 138648 Singapore; 5 The School of Biomedical Sciences, The University of Queensland, Queensland, Brisbane Australia

## Abstract

Yes-associated protein (YAP) is a mechanosensor protein and a downstream effector of the Hippo kinase pathway, which controls organ growth, cell proliferation, survival, maintenance and regeneration. Unphosphorylated YAP translocates to the nucleus where it acts as a cofactor of primarily the TEAD transcription factors to activate target gene transcription and cell proliferation. Perturbed YAP activation results in tumorigenesis. The pathways downstream of activated YAP that drive cell proliferation remain relatively unexplored. In this study, we employed YAP2-5SA-∆C transgenic mice, which overexpress a mildly activated YAP mutant protein in basal keratinocytes leading to increased proliferation of the epidermal stem/progenitor cell populations. We performed massively-parallel sequencing of skin biopsy mRNA (RNA-Seq) and found dysregulation of 1491 genes in YAP2-5SA-∆C skin, including many with roles in cell activation and proliferation. Furthermore, we found that 150 of these dysregulated genes harbored YAP/TEAD binding motifs in the 3′ UTR, suggesting that these may be direct YAP/TEAD target genes in the control of epidermal regeneration. Further validation and functional characterization assays identified *Plau* and *Tgfbr3* as prime candidate genes that may be activated by epidermal YAP activity in the mouse skin in vivo to promote keratinocyte proliferation. This study provides novel insights into the mechanisms regulated by YAP that control tissue homeostasis, and in particular in conditions where YAP is aberrantly activated such as in neoplastic and regenerative skin disease.

## Background

Tissue growth during embryonic development and during postnatal regeneration are intricately coordinated processes that are perturbed during regenerative disease. Oncoprotein Yes-associated protein (YAP) is a critical and highly conserved regulator of organ size and tumorigenesis. As a transcriptional coactivator, YAP shuttles between cytosol and the nucleus. In the cytosol, YAP is inactive. In the nucleus, it interacts with the TEAD transcription factors to activate target gene transcription and cell proliferation^1,2^.

YAP activity is generally known to be controlled by the conserved Hippo kinase pathway, which ultimately phosphorylates and inactivates YAP by cytosolic retention^1^. However, recent pioneering work has demonstrated that local mechanical cues play a major overarching role in the regulation of YAP nucleocytoplasmic shuttling^3,4^. The earliest evidence of this comes from a study that shows that YAP nuclear localization and activity are inversely correlated with cell density [7]. Moreover, recent studies have shown that cell shape and size, cell-cell, and cell-matrix interactions play a major role in regulation of YAP activity^3,4^. In fact, these mechanical cues dominate over the activity of the core Hippo kinase cassette in the regulation of YAP activity ^3,4^.

Our understanding of the upstream mechanisms controlling YAP activity has advanced considerably in the last ten years, yet our understanding of what occurs downstream of YAP to drive cell proliferation and to maintain tissue homeostasis remains relatively limited. This study investigated the genes regulated by epidermal YAP activity to promote keratinocyte proliferation in the mouse skin using the YAP2-5SA-∆C transgenic mouse model. These mice express a mildly dominant active YAP mutant protein in basal Keratin5/14-positive keratinocytes, leading to severe skin abnormalities, including epidermal hyperplasia, progressive dorsal alopecia due to abnormal hair follicles, loss of whiskers and dry, scaly skin^5^. These abnormalities are caused by increased proliferation of the basal stem/progenitor cell populations displaying high nuclear β-catenin and GLI2 activity^5–8^. There were however no signs of tumour formation in the skin of YAP2-5SA-∆C mice^5^. Therefore, this transgenic mouse model allows for investigation of the molecular changes downstream of activated YAP to promote cell proliferation in vivo, but not leading to tumorigenesis.

We performed whole transcriptome sequencing of skin tissue of YAP2-5SA-∆C transgenic mice to obtain insights into the global changes of gene expression in the mouse skin that drive keratinocyte proliferation in response to epidermal YAP activity, and to identify putative YAP/TEAD direct target genes in epidermal regeneration. *Plau*, the gene encoding Plasminogen Activator, Urokinase, and *Tgfbr3* were identified as prime candidate genes that may be activated in response to epidermal YAP activity to promote keratinocyte proliferation in the epidermis.

## Results

### RNA-Seq analysis shows widespread gene dysregulation in the YAP2-5SA-∆C skin

**Fig. 1:**
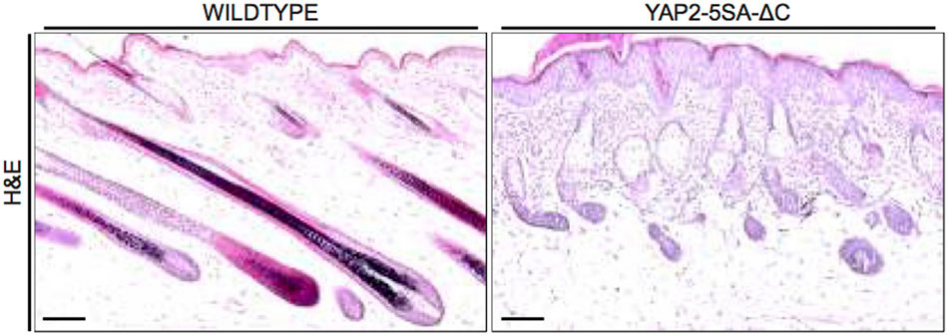
YAP2-5SA-ΔC mouse skin histology. Hematoxylin and eosin staining showing the skin histology of wildtype (**a**) and YAP2-5SA-ΔC (**b**) mice. Scale bars = 20 µm

To obtain insights into the global changes of gene expression in the mouse skin in response to epidermal YAP activity, we performed whole transcriptome sequencing of skin tissue of YAP2-5SA-∆C transgenic mice. RNA was extracted from dorsal neck skin biopsies of three P33 female YAP2-5SA-∆C mice (TG) showing the early onset of the externally visible phenotype^5^, and of three female wild type littermate mice (WT). Histology analyses confirmed epidermal hyperplasia in YAP2-5SA-∆C skin consistent with previous reports (Fig. 1)^5^. The six RNA-Seq libraries were sequenced on the Illumina NextSeq platform to produce approximately 30 million, 75 nucleotide paired-end reads per sample. Differential expression analysis was performed using the Bioconductor packages, edgeR^9^ and limma (Voom)^10^ as detailed in Methods.

**Fig. 2:**
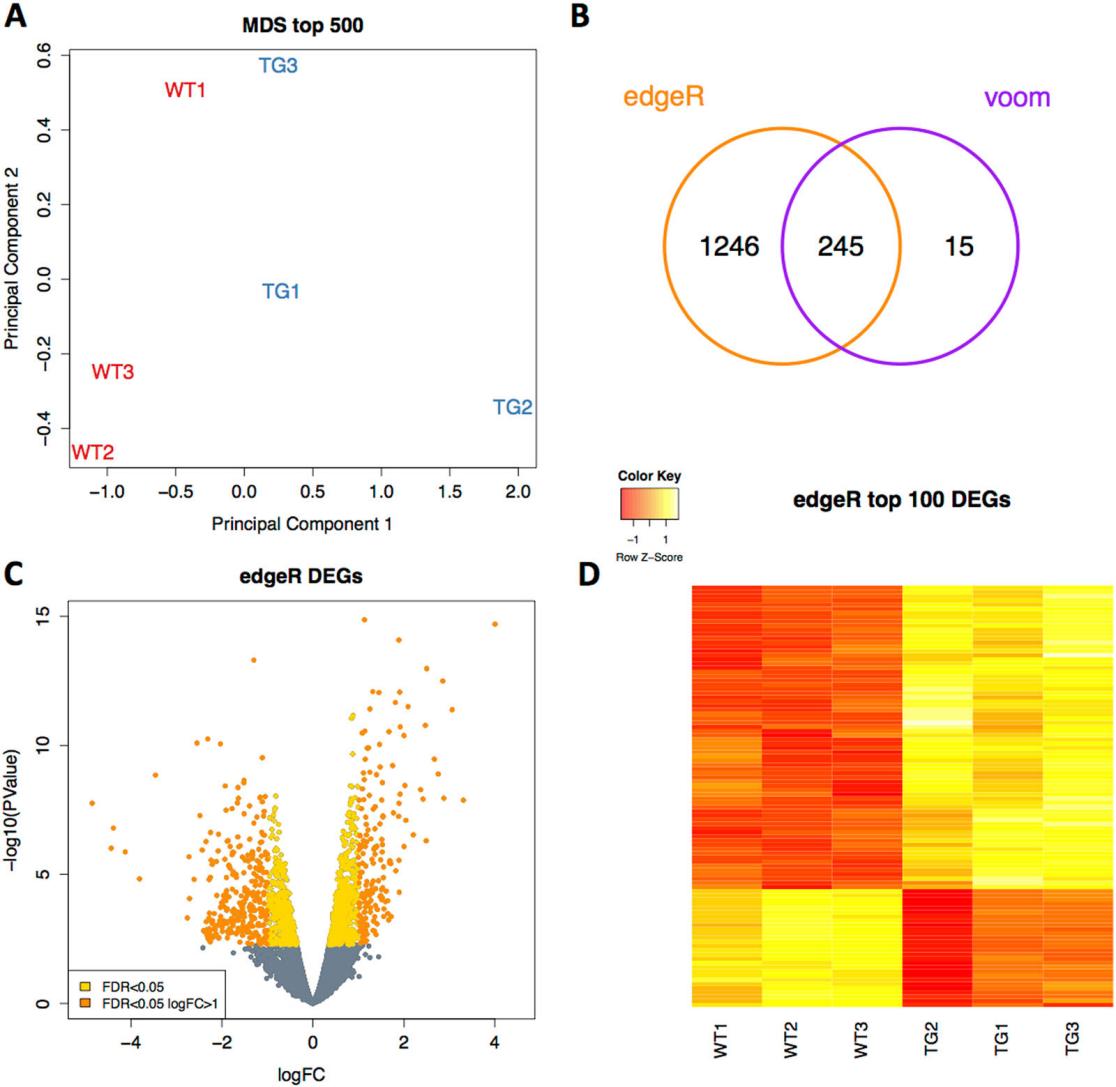
RNA-Seq differential expression. **a** Multidimensional scaling (MDS) plot showing separation of wild type (WT) and YAP2-5SA-ΔC (TG) samples. **b** Venn diagram of differentially expressed genes found by edgeR and voom (FDR < 0.05). **c** Volcano plot showing log fold change (logFC) on the x-axis and –log10 (*P* value) on the y-axis of gene expression alterations found using edgeR. Genes with FDR > 0.05 yellow and orange dots. Genes with greater than 2 fold change (logFC > 1) orange dots. **d** Heatmap showing change in gene expression across all WT and YAP2-5SA-ΔC (TG) samples for the top 100 differentially expressed genes (decreased expression (red) increased expression (yellow)

The wildtype and YAP2-5SA-∆C transcriptomes could be distinguished along the first principal component using a multidimensional scaling (MDS) plot (Fig. 2a). Differential expression analysis using edgeR identified 1491 differentially expressed genes (DEGs) at an FDR < 0.05. Using voom we found a more conservative set of 260 DEGs of which 94% (245/260) were also included in the edgeR results (Fig. 2b). A volcano plot of the edgeR results shows a similar number of up-regulated and down-regulated genes with a tendency towards the most statistically significant DEGs being up-regulated (Fig. 2c). Clustering analysis of the top 100 DEGs (edgeR; ranked by statistical significance) also demonstrates this tendency for up-regulation in the YAP2-5SA-∆C skin (Fig. 2d), in line with the role of YAP as transcriptional activator ^11^.

The DEGs were sorted by statistical significance (lowest FDR), and details of the differentially expressed genes can be found in Supplementary Table S1. Among upregulated genes are those that encode keratinocyte-specific intermediate filament proteins including *Krt6b/16*, (edgeR: FC = 7.23, FDR = 3.13E-10), which is typically upregulated in hyperproliferative and inflamed epidermis^12^. Also, the expression of *Krt14* (edgeR: Fold Change (FC) = 1.94, FDR = 0.085), which marks the expanded basal epidermal layer^5,13^, and *Inhba*2 (edgeR: FC = 1.57, FDR = 0.07) and *Cyr61*^14^ (edgeR*:* FC *=* *1.28, FDR* *=* *0.15)*, known direct targets of YAP, appear to be expressed at higher levels in YAP2-5SA-∆C skin although not reaching our statistical cut-off. In contrast, expression of YAP direct target *Ctgf*2 (edgeR: FC = −1.76, FDR = 2.07E-06) was downregulated in YAP2-5SA-∆C skin.

**Fig. 3:**
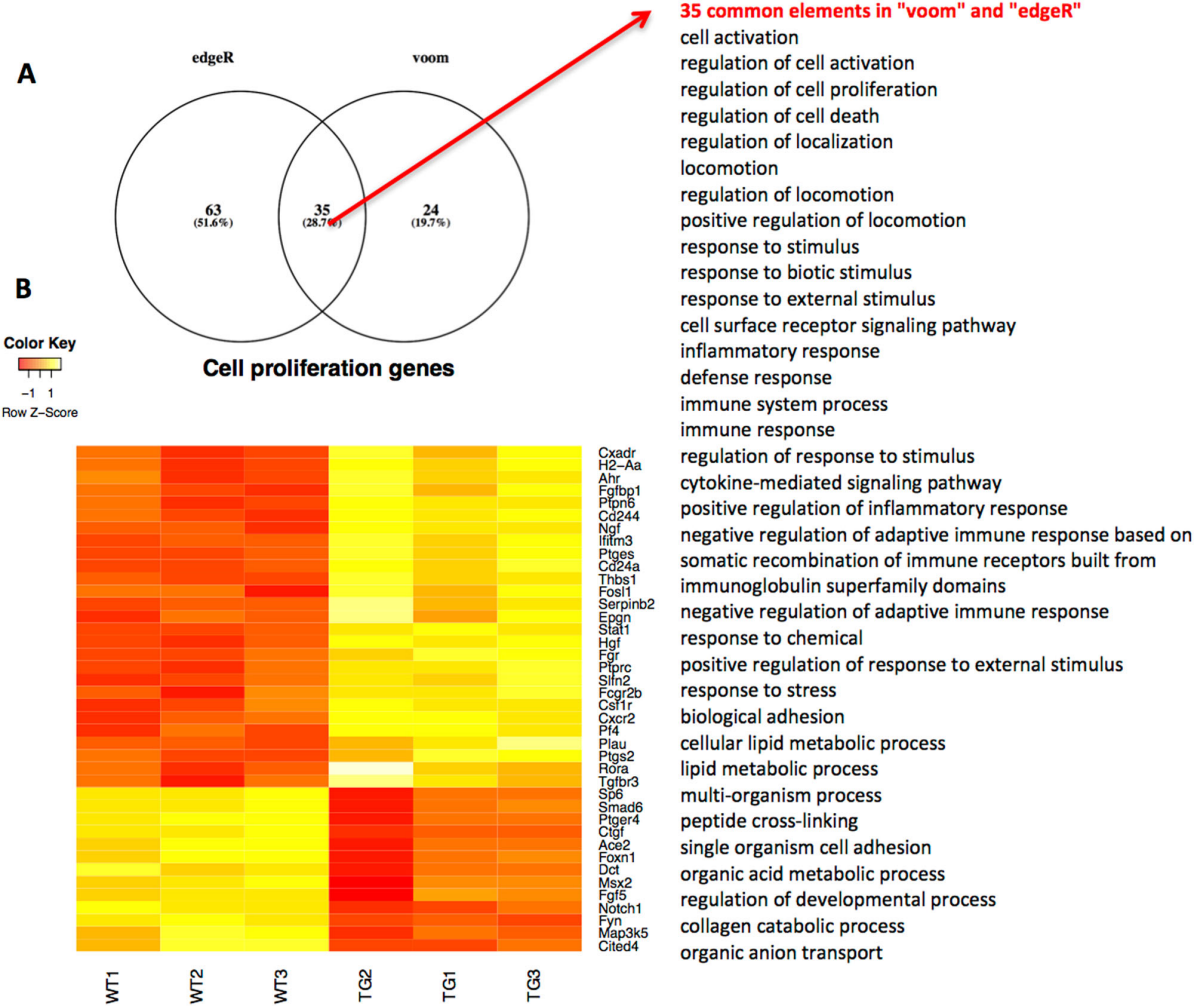
Enriched gene ontology terms in the differentially expressed genes found by RNA-Seq. **a** Venn diagram of enriched gene ontology terms (Biological Process) in the edgeR and voom differentially expressed gene set. Enriched gene ontology terms were found using GOrilla and REVIGO. **b** Heatmap showing change in gene expression across all WT and YAP2-5SA-ΔC (TG) samples in differentially expressed genes involved in cell proliferation (decreased expression (red) increased expression (yellow)

To identify the main biological processes regulated by YAP, we analysed the DEGs identified by voom and edgeR using the GOrilla and REVIGO tools. This identified 35 common gene ontology terms, which included cell proliferation, cell death, locomotion, immune processes and lipid metabolism (Fig. 3a). Interestingly, most of the genes related to cell proliferation were upregulated in YAP2-5SA-∆C skin (Fig. 3b).

**Fig. 4:**
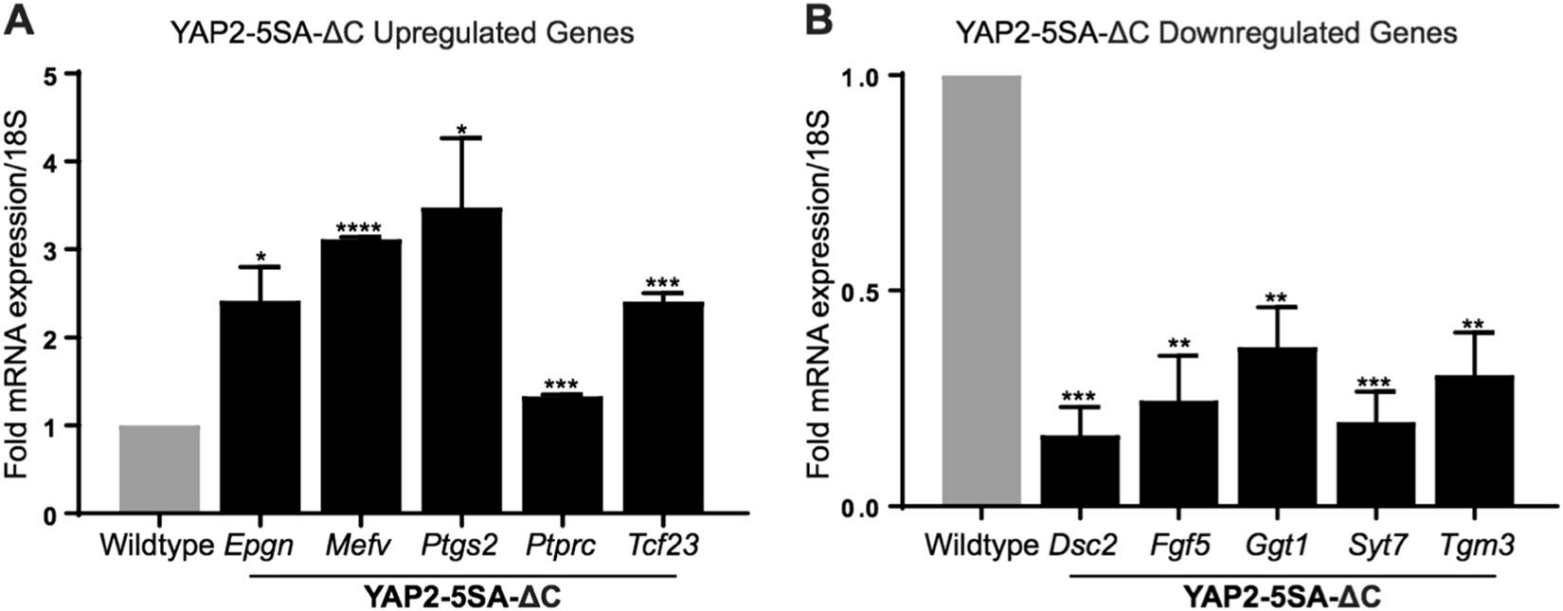
YAP2-5SA-ΔC transcriptomics validation by qRTPCR. Quantitative RT-PCR analyses showing the expression levels of (**a**) 5 upregulated and (**b**) 5 downregulated genes in YAP2-5SA-ΔC skin relative to 18 S RNA, and normalized to wildtype levels. Error bars represent SEM (*P value ≤ 0.05; **P value ≤ 0.002; ***P value ≤ 0.001; ****P value ≤ 0.0001)

In order to experimentally validate the transcriptomics results, we selected 5 up- and 5 downregulated genes from the edgeR analysis, and we performed quantitative real time RT-PCR assays on the same cohort to determine their expression levels in wildtype and YAP2-5SA-∆C skin biopsies. The genes selected for testing have functional annotations involving regulation of cell proliferation and cell cycle (*Fgf5, Ptprc, Ptgs2, Epgn*), development (*Tgm3*, *Tcf23*), inflammatory response and repair (*Syt7*, *Ggt1*, *Mefv*) and cell adhesion (*Dsc2*) (Supplementary Table S2). Results of the analyses confirmed significantly increased expression of *Epgn*, *Mefv*, *Ptgs2*, *Ptprc* and *Tcf23* (Fig. 4a; all *P* < 0.05, *N* = 3), and significantly decreased expression levels of *Dsc2*, *Fgf5*, *Ggt1*, *Syt7*, and *Tgm3* (Fig. 4b; all *P* < 0.005, *N* = 3), in line with the transcriptomics screen outcome.

Altogether, these data show that the transcriptomics outcome is a robust platform to investigate the mechanisms regulated downstream of epidermal YAP activity to control regeneration of the skin.

### Identification of YAP/TEAD binding motifs in the 3′ UTRs of 150 differentially expressed genes

**Fig. 5:**
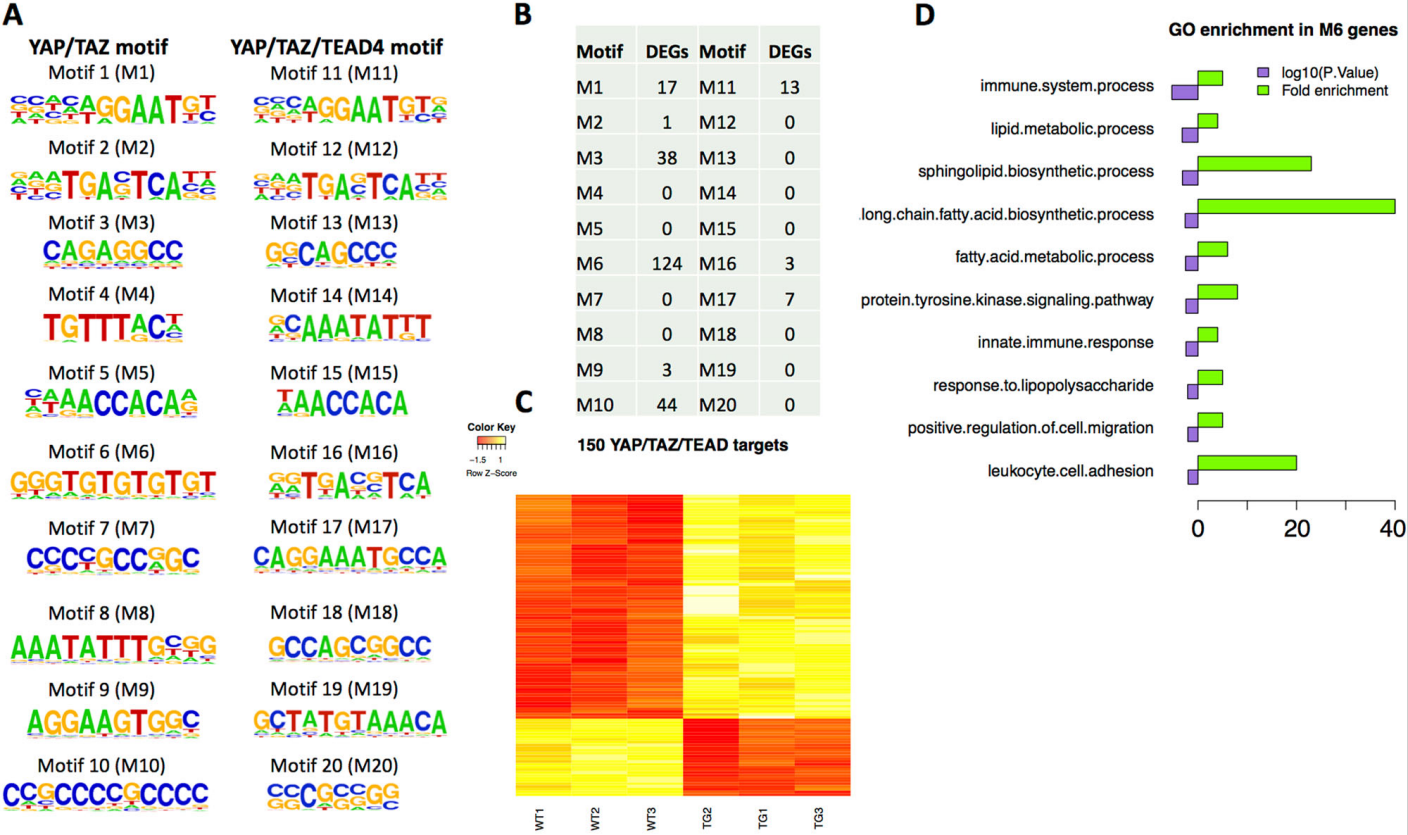
Genes with YAP/TAZ/TEAD motifs in the 3′ UTR. **a** 20 YAP/TAZ/TEAD motifs identified in reference^15^. **b** Number of differentially expressed genes found by voom which have one of the 20 motifs in the 3′ UTR. **c** Heatmap showing change in gene expression across all WT and YAP2-5SA-ΔC (TG) samples in 110 differentially expressed genes which harbor the YAP/TAZ/TEAD motifs (decreased expression (red) increased expression (yellow). **d** Top 10 Enriched Gene ontology terms (Biological Process) found using the DAVID tool in the set of 124 genes found to harbor the M6 motif in the 3′ UTR region

Zanconato et al. recently performed ChIP-seq studies to identify gene loci bound by YAP/TAZ/TEAD in breast cancer cells. Motif analyses identified 20 potential YAP/TAZ and YAP/TAZ/TEAD binding motifs^15^. To identify putative YAP/TEAD direct target genes in the epidermis, we investigated whether these motifs appeared in the regulatory regions of the differentially expressed genes identified in our transcriptomics study. To narrow the scope of this analysis we used the more conservative set of differentially expressed genes found by voom. We specifically interrogated their 3’ untranslated regions (UTRs), since Zanconato et al. found that these motifs tended to occur in enhancer regions rather than promoter regions. Furthermore, two other *bona fide* YAP/TEAD direct target genes *Sox2* and *Snai2* in cardiomyocyte progenitor cells were recently found to harbor YAP/TEAD binding motifs in their 3′ UTRs^16^. The statistical significance of the presence of a binding motif in our gene set was assessed using FIMO^17^. To assess the reliability of our analysis, we first identified motifs in genes, which are known YAP/TEAD target genes *Ctgf*,2 *Cyr61*^14^, *Gli2*^18^ and *Inhba*.2 We indeed found that these genes contained one or more of the identified motifs in the 3′ UTR (Supplementary Table S3). For ease of reference we have ordered and named these motifs in the same order as they appear in Zanconato et al. Supplementary Table 1, Motifs 1- 20 (M1-M20) as shown in Fig. 5a. Next, we established that 150 of the 260 differentially expressed genes identified in the voom analysis had one or more binding motifs at a statistically significant level (*P* value < 0.0001, *q* value < 0.2; Fig. 5b and Supplementary Table S3). A heatmap of these 150 genes (Fig. 5c) shows that a majority are up-regulated in YAP2-5SA-∆C skin, in line with the function of YAP as transcriptional activator. Notably, the M6 motif was detected in the 3′ UTR of 124 genes, which were found to be enriched for biological processes involving lipid synthesis as well as immune processes (Fig. 5d). At the other extreme, the motifs, M4, M7, M8, M12, M13, M14, M15, M19 and M20 were not represented in the tested gene set. Overall, these analyses have identified 150 candidate direct YAP/TAZ/TEAD target genes that may be involved in regulating epidermal regeneration.

### Identification of *Plau* and *Tgfbr3* as candidate genes that may be activated by epidermal YAP activity to promote keratinocyte proliferation in the mouse skin

The Hippo/YAP signaling pathway regulates cell proliferation and organ size in various tissues^1,19,20^. However, the mechanisms downstream of YAP that drive cell proliferation remain largely unknown. To reveal which of the differentially expressed genes identified in the hyperproliferative skin of YAP2-5SA-∆C mice may directly or indirectly be activated by YAP in the epidermis to drive keratinocyte proliferation, we selected 17 genes for further validation studies based on gene ontology terminology. These included genes with known roles in cell proliferation (GO:0008283: *ACE2*, *CITED4*, *FGF5*, *FOXN1*, *MSX2*, *SERPINB2*, *EPGN*, *FGR*, *FOSL1*, *HGF*, *PLAU*, *PTGS2*, *RORA*, *TGFBR3*), or in the regulation of cell death (GO:0006355: *BMP4*, *GADD45A* and *PAWR*). All of these genes were differentially expressed in either or both the edgeR and voom analyses at an FDR less than 0.1 (Supplementary Table S4).

**Fig. 6:**
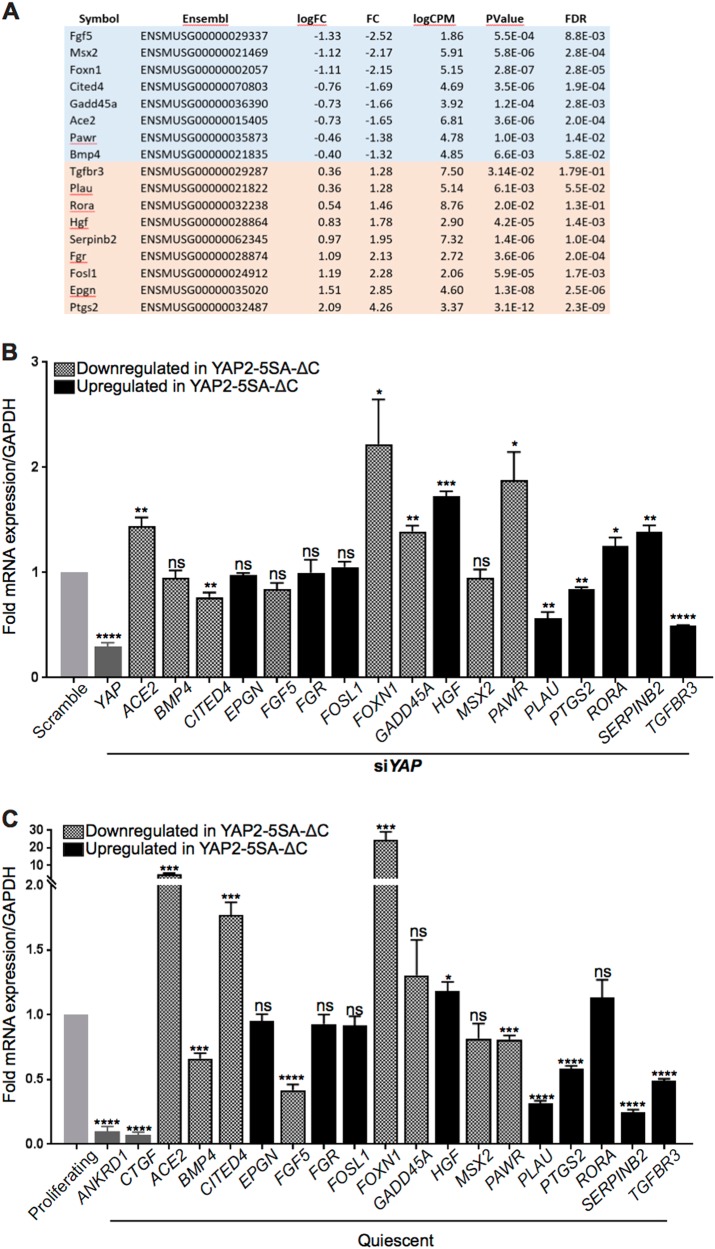
Identification of candidate genes activated by epidermal YAP activity in the regenerating mouse skin. Quantitative RT-PCR analyses showing the expression levels a set of 17 identified differentially expressed genes in (**a**) RNA-Seq results obtained from the edgeR analysis for the 17 genes (**b**) YAP depleted HaCaT keratinocytes relative to *GAPDH* RNA, and normalized to levels in Scramble control transfected keratinocytes, and in (**c**) quiescent HaCaT keratinocytes relative to *GAPDH* RNA, and normalized to levels in proliferating keratinocytes, respectively. Error bars represent SEM (*P value ≤ 0.05; **P value ≤ 0.002; ***P value ≤ 0.001; ****P value ≤ 0.0001)

We first measured their expression levels in response to YAP activity in keratinocytes grown in vitro. We inactivated *YAP* in proliferating HaCaT keratinocytes grown in vitro and quantified gene expression levels by quantitative real time RT-PCR assays. These confirmed significantly decreased expression of *YAP* (*P* < 0.0005; *N* = 3) in si*YAP vs*. siScramble-transfected HaCaT keratinocytes (Fig. 6a). Furthermore, expression of *CITED4* (*P* < 0.007; *N* = 3), *PLAU* (*P* < 0.003; *N* = 3), *PTGS2* (*P* < 0.002, *N* = 3) and *TGFBR3* (*P* < 0.0001; *N* = 3) were also decreased, whereas expression of *ACE2* (*P* < 0.008; *N* = 3), *FOXN1* (*P* < 0.05; *N* = 3), *GADD45A* (*P* < 0.0025; *N* = 3), *HGF* (*P* < 0.00015; *N* = 3), *PAWR* (*P* < 0.04; *N* = 3), *RORA* (*P* < 0.04; *N* = 3) and *SERPINB2* (*P* < 0.005; *N* = 3) were increased (Fig. 6a). *BMP4*, *EPGN*, *FGF5*, *FGR*, *FOSL1* and *MSX2* expression levels were unchanged (*N* = 3; Fig. 6a). Altogether, these data showed that *PLAU*, *PTGS2*, and *TGFBR3* expression are positively regulated, whereas *ACE2*, *FOXN1*, *GADD45A*, *PAWR* expression are negatively regulated by YAP activity both in proliferating keratinocytes in the mouse skin in vivo, and in HaCaT keratinocytes grown in vitro.

Next, to investigate the possible role of these 17 candidate YAP-regulated genes in keratinocyte proliferation, we conducted qRT-PCR analyses using RNA extracted from high-dense and quiescent *vs*. sparse and proliferating HaCaT keratinocytes. Quiescent keratinocytes showed significantly lower expression levels of known direct target genes of YAP, *ANKRD1* (*P* < 0.0001; *N* = 3) and *CTGF* (*P* < 0.0001; *N* = 3) confirming decreased YAP transcriptional activity (Fig. 6b), consistent with previous data^21^. Furthermore, expression levels of *BMP4* (*P* < 0.0002; *N* = 3), *FGF5* (*P* < 0.0001; *N* = 3), *PAWR* (*P* < 0.0006; *N* = 3), *PLAU* (*P* < 0.0001; *N* = 3), *PTGS2* (*P* < 0.0001; *N* = 3), *SERPINB2* (*P* < 0.0001; *N* = 3) and *TGFBR3* (*P* < 0.0001, *N* = 3) were lower in quiescent *vs*. proliferating keratinocytes, whereas expression levels of *ACE2* (*P* < 0.0001; *N* = 3), *CITED4* (*P* < 0.0003; *N* = 3), *FOXN1* (*P* < 0.001; *N* = 3) and *HGF* (*P* < 0.02; *N* = 3) were higher (Fig. 6b). *EPGN*, *FGR*, *GADD45A*, *MSX2* and *RORA* expression levels remained unchanged (*N* = 3, Fig. 6b). The expression levels of *ACE2*, *CITED4*, *FOXN1*, *PLAU*, *PTGS2*, *SERPINB2* and *TGFBR3* in quiescent *vs*. proliferating HaCaT keratinocytes correlated with those observed in the hyperproliferative skin of YAP2-5SA-ΔC *vs*. wildtype littermate mice as determined by our transcriptomics screen.

**Fig. 7:**
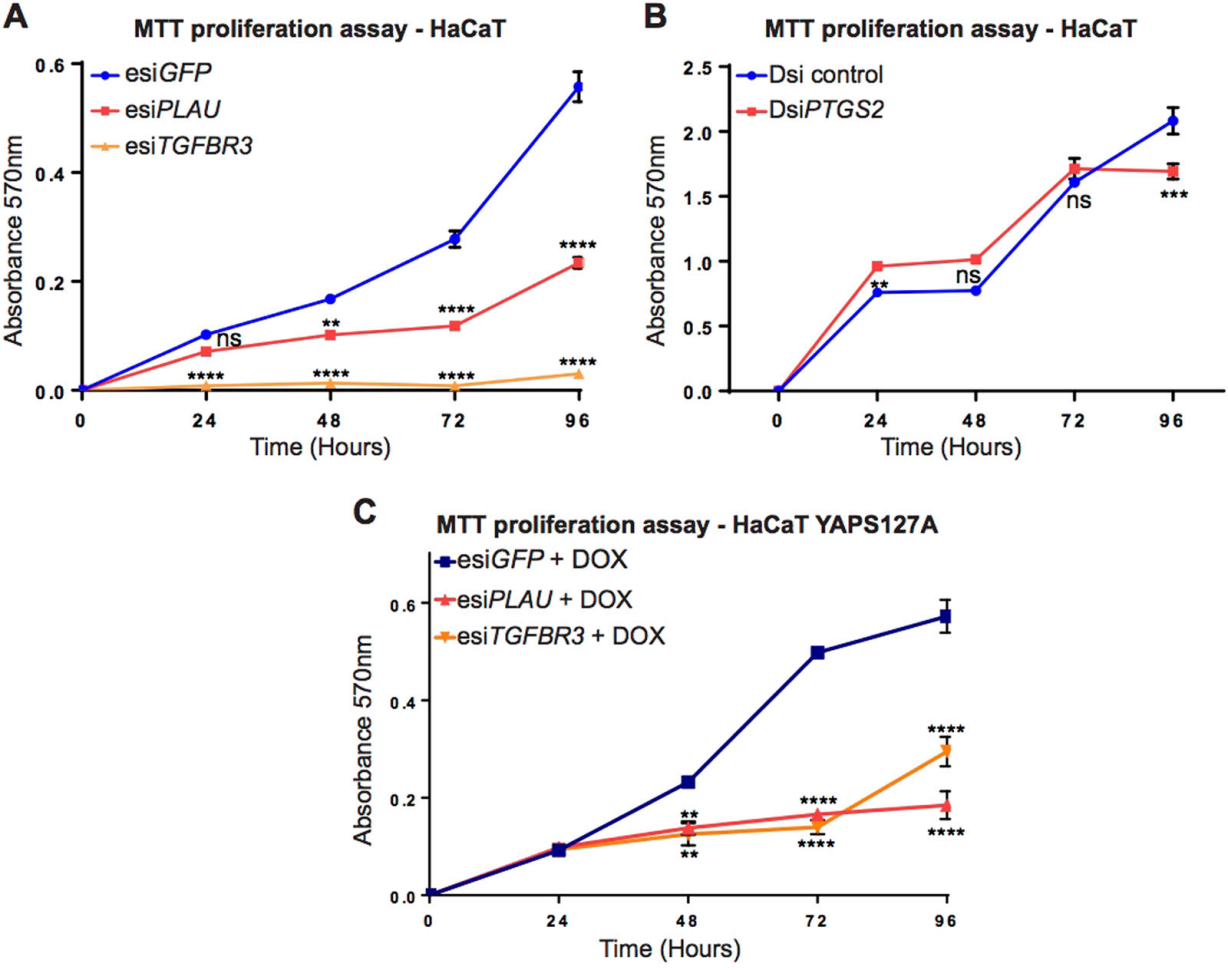
*TGFBR3* or *PLAU* depletion results in reduced keratinocyte proliferation rates. MTT proliferation assays using HaCaT keratinocytes transfected with (**a**) esi*GFP*, esi*PLAU*, esi*TGFBR3*, (**b**) DsiControl and Dsi*PTGS2* RNA (*N* = 3) or (**c**) esi*GFP*, esi*PLAU*, esi*TGFBR3* in YAPS127A Doxyccycline induced HaCaT keratinocytes (*N* = 3)

Altogether, *PLAU*, *PTGS2* and *TGFBR3* responded most consistently as candidate genes that may be positively regulated by the transcriptional coactivator YAP in the epidermis to promote keratinocyte proliferation both in vitro and in the mouse skin in vivo. We therefore next assessed their roles in driving HaCaT keratinocyte proliferation in vitro in MTT proliferation assays. We detected significantly reduced proliferation in esi*TGFBR3* and esi*PLAU vs*. esi*GFP*-transfected HaCaT keratinocytes from 24 and 48 h post-transfection onwards, respectively (Fig. 7a, *P* < 0.0001; *N* = 3). Proliferation of esi*PTGS2*-transfected cells was relatively unaffected, and displayed only mildly decreased proliferation rates at 96 h after transfection, the latest time point measured (Fig. 7b).

To assess if YAP drives HaCaT keratinocyte proliferation in a PLAU and TGFBR3-dependent manner, we generated an inducible HaCaT keratinocyte cell line that expresses dominant active YAPS127A upon doxycycline (DOX) treatment. We detected reduced cell proliferation in esi*PLAU* and esi*TGFBR3 vs*. esi*GFP*-transfected YAPS127A expressing HaCaT keratinocytes treated with DOX (Fig. 7c). These data confirm that PLAU and TGFBR3 act as downstream factors that mediate the effect of YAP on HaCaT keratinocyte proliferation in vitro.

**Fig. 8:**
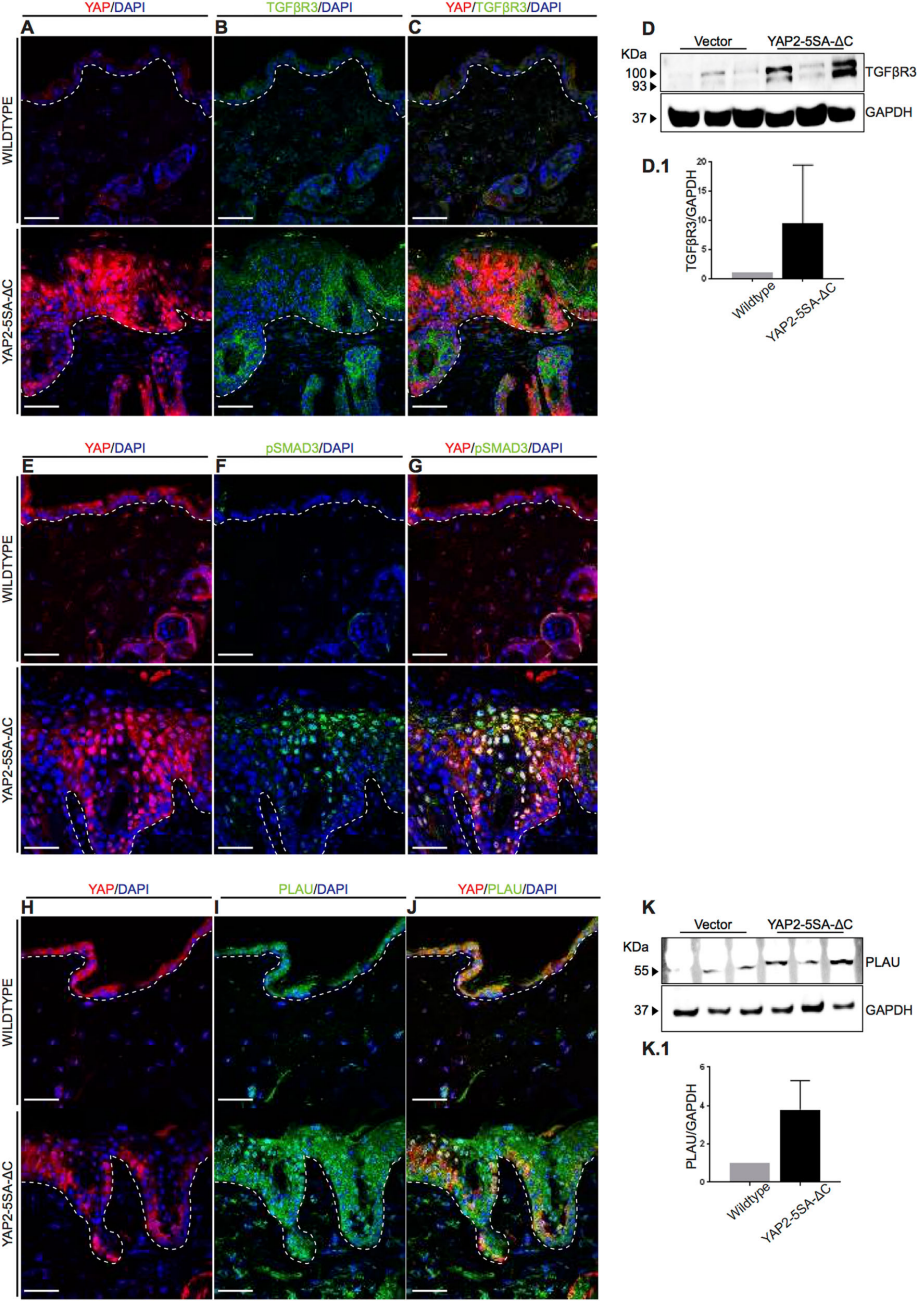
TGFBR3 and PLAU protein expression in wildtype and YAP2-5SA-ΔC mouse skin. **a-c** Immunofluorescence staining of wildtype (top) and YAP2-5SA-ΔC^54^ mouse skin showing TGFBR3 (green) and YAP (red) protein expression in YAP2-5SA-ΔC epidermis. **d** Western blots detecting TGFBR3 (100 KDa) and GAPDH (37 KDa) in protein lysates of wildtype and YAP2-5SA-ΔC skin (N = 3). (**d**.1) Quantification of fold-change TGFBR3 protein expression levels normalized to GAPDH. **e**-**g** Immunofluorescence staining of wildtype (top) and YAP2-5SA-ΔC^54^ mouse skin showing phospho-SMAD3 (green) and YAP (Red) protein expression. **h**-**j** Immunofluorescence staining of wildtype (top) and YAP2-5SA-ΔC^54^ mice skin showing PLAU (green) and YAP (red) protein expression in YAP2-5SA-ΔC epidermis. **k** Western blots detecting PLAU (55 KDa) and GAPDH (37 KDa) in protein lysates of wildtype and YAP2-5SA-ΔC skin (*N* = 3). **k**.1 Quantification of fold-change PLAU protein expression levels normalized to GAPDH. Scale bars = 20 µm

Lastly, we investigated TGFBR3 and PLAU protein expression levels in skin biopsies of YAP2-5SA-ΔC mice. We confirmed increased TGFBR3 protein expression in the epidermis of YAP2-5SA-ΔC compared to wildtype mice by immunofluorescence and western blot analyses (Figs. 8b; 8d & 8d.1). In addition, we also detected increased nuclear localization of phospho-SMAD3 in the keratinocytes (Fig. 8f), indicating increased TGFβ signaling activity in the epidermis of YAP2-5SA-ΔC mice. Furthermore, we also detected increased PLAU protein expression throughout the YAP2-5SA-ΔC epidermis (Figs. 8i; 8k & 8k.1). Altogether these data identify *TGFBR3* and *PLAU* as prime candidate genes that may be positively regulated by epidermal YAP activity to promote keratinocyte proliferation in mouse skin.

## Discussion

In this study, we identified the global changes of gene expression in the mouse skin in response to epidermal YAP activity by sequencing the skin RNA of YAP2-5SA-ΔC mice, which overexpress a mildly activated YAP mutant protein in basal cutaneous keratinocytes, resulting in severe epidermal hyperplasia due to an expanded epidermal stem/progenitor cell population^5^. We identified 1491 differentially expressed genes that are regulated by epidermal YAP in the hyperproliferative YAP2-5SA-ΔC skin. The main biological processes regulated by YAP in YAP2-5SA-ΔC skin included cellular response to stimulus, cell proliferation and cell adhesion, which is consistent with previously published transcriptomics analyses using in vitro grown keratinocytes with modified YAP activity^14,22,23^. 150 of the high-reliability subset of 260 differentially expressed genes identified by voom, harbour YAP/TEAD binding motifs in their 3′ UTRs, making them potential YAP/TEAD direct target genes in the control of keratinocyte proliferation. Our functional characterization assays subsequently identified *Tgfbr3* and *Plau* as prime candidate genes that may be positively regulated by YAP activity in the epidermis to promote keratinocyte proliferation in the mouse skin.

RNA-Seq allows detection of differential expression over a broader dynamic range than microarray thus allowing the detection of differences in lowly and highly expressed genes^24^. We have therefore been able to find a larger set of genes undergoing YAP mediated expression change than seen in earlier studies which used microarray technology^14,25^. We performed our RNA-Seq analysis using edgeR and also limma (voom), which found a more conservative list of differentially expressed genes. This finding is consistent with previous observations that limma (voom) gives a more conservative number of differentially expressed genes whilst achieving high precision^26^. We used this more conservative list of differentially expressed genes to undertake an initial search for YAP/TEAD motifs previously identified by Zanconato et al. in enhancer areas of target genes^15^. On this basis, we decided to explore the 3’ UTR of genes in our high confidence subset of differentially expressed genes, as this region has been found to have a potent enhancer function including recruitment of enhancing binding factors in other systems^27^, and in particular because two other YAP/TEAD direct target genes *Sox2* and *Snai2* have recently been found to have YAP/TEAD binding motifs in the 3′ UTR^16^. To undertake this search we employed the widely used FIMO tool^17^. We found that M6 was most highly represented in the differentially expressed genes tested (*n* = 124), followed by M10 (*n* = 44) and then M3. It is interesting to note that Zanconato et al. found that these three binding motifs were most similar to the target regions of the zinc finger transcription regulators, Gm397_2, Sp1(Zf) and ZFX(Zf) respectively (ref ^15^ Supplementary Table 1). Not all motifs were detected; M4, M5, M7, M8, M12, M13, M14, M15, M18, M19 and M20 were not represented in the subset of differentially expressed genes tested. This is an indication that those motifs detected and highly represented in our set of genes may be of biological significance. An analysis of gene ontology terms enriched in the subset of 124 genes with the M6 motif showed that these genes can be distinguished by their role in lipid biosynthesis, including sphingolipid biosynthesis. Notably, sphingolipid dysfunction has been associated with dry skin conditions such as atopic dermatitis and disrupted barrier function of the skin ^28,29^.

The transforming growth factor beta (TGF-β) superfamily members have pleiotropic functions regulating proliferation, differentiation, stem-cell maintenance, regeneration, extracellular matrix production, angiogenesis and immune response^30^. Activation of the TGF-β signaling pathway results in the phosphorylation and nuclear translocation of the SMAD transcription factors to orchestrate transcription^31^. In our study, we demonstrated that TGFBR3 promotes keratinocyte proliferation in vitro. This observation is in line with a previous study that showed that *TGFBR3* depletion results in reduced colony formation in 368T1 and 482N1 cells^32^, and in decreased cell proliferation and increased cell apoptosis in *Tgfbr3* loss-of-function mice during palatal shelf development^33^. Furthermore, we find that epidermal YAP activity results in increased TGFBR3 expression and increased nuclear phospho-SMAD3 in the hyperproliferative epidermis of YAP2-5SA-ΔC mice. Interestingly, YAP and the TGF-β signaling pathways have previously been shown to undergo intimate regulatory interactions in other systems. Nuclear translocation of the SMAD2/3 complex was shown to be dependent on YAP activation and cell density^34^^35^, and may occur in response to degradation of the Hippo pathway scaffold protein RASSF1A upon TGF-β stimulation^36^. Indeed, nuclear YAP/TAZ/SMAD complexes were recently also detected in sparse keratinocytes grown in vitro in response to TGF-β stimulation, but less so in high dense keratinocytes^37^, which is in line with our results. However, the underlying molecular nature of the regulatory interaction between YAP and *Tgfbr3* remains to be established.

Plasminogen activator, urokinase (PLAU) is a serine protease that has a key role in the regulation of cell migration, proliferation and adhesion processes in cancer and inflammation^38–42^ and in muscle, liver and axonal regeneration^43,44^. It binds its receptor PLAUR to initiate a proteolytic cascade that converts plasminogen to plasmin. Here we show that PLAU expression is positively regulated by YAP activity in keratinocytes in the skin of the YAP2-5SA-ΔC mice and in HaCaT keratinocytes cultured in vitro. Furthermore, we demonstrate a significant reduction of proliferation rates in esi*PLAU*-transfected HaCaT keratinocytes compared to esiRNA control, in line with previous studies showing reduced keratinocyte proliferation rates in the skin of *Plau* loss of function mice^45^. The hyperplastic YAP2-5SA-ΔC skin transcriptome analysis also showed *Plaur* upregulation as well as an increase in *Plau* which was just under over statistical cut-off (FDR = 0.055), which suggests that epidermal YAP activity may promote both PLAU and PLAUR expression to promote keratinocyte proliferation. In addition, we found that the 3′ UTR of PLAU, PLAUR and TGFBR3 contain YAP/TEAD binding motifs as identified by Zanconato et al^15^. (Supplementary Table 3). Future studies will confirm if the epidermal hyperplasia in the YAP2-5SA-ΔC mice is caused by YAP activation and its direct or indirect regulation of *Plau* and *Plaur* and, in consequence, the activation of the PLAU/PLAUR signaling.

Previous studies have shown that YAP is activated in neonatal epidermis and in wound healing, but that it is not per se involved in the maintenance of normal homeostasis of postnatal skin (see for instance 20, 21). In our studies, YAP is artificially activated in the murine epidermis, and therefore our data do not conclusively show that YAP drives *Plau* and *Tgfbr3* expression in normal skin homeostasis in mice in vivo. Unfortunately, this can also not be determined with certainty at this stage, as mice in which YAP is inactivated in the basal epidermis are not postnatally viable^14,46^. However, the regulatory interaction that we identified may be biologically relevant in the context of neoplastic skin or other regenerative skin disorders, where YAP is aberrantly activated in keratinocytes, similar to in the YAP2-5SA-ΔC mouse line. In line with this, we found a nearly 4-fold increase in genes common with those identified in the squamous cell carcinoma-derived SCC13 cell line *vs*. the Normal Human Keratinocyte cells as reported by^23^ (Supplementary Table S7). These common genes were enriched for gene ontology terms involving signalling and included *Plaur* and *Tgfbr3* (but not *Plau* and *Tgfbr3*), and with cell adhesion consistent with the findings of Elbediwy et al. 2016^22^. Therefore, this study provides important novel insights into the genetic mechanisms controlled by YAP that may be perturbed in neoplastic and regenerative skin disease.

## Methods

### Animals

All animal experimental procedures on YAP2-5SA-ΔC mice and wildtype littermates were conducted under protocols approved by the UNSW Australia’s Animal Care and Ethics Committee Unit, and in compliance with the National Health and Medical Research Council’s Australian code of practice (8th edition, 2013).

### Cell culture, transfections, and MTT assays

HaCaT immortalized keratinocytes were maintained in DMEM/F-12 (Sigma, D8062), supplemented with 10% FBS (Gibco, 10437-028) and 1X Penicillin-Streptomycin (Gibco, 15140-122) in a 5% CO2 incubator at 37 °C. Transient transfections were performed using Lipofectamine3000 (Thermo Fisher Scientific, L3000015) according to manufacturer’s instructions. *YAP* knockdown was performed using MISSION® Universal and *YAP* siRNA (Sigma). *TGFBR3* and *PLAU* knockdown was performed using MISSION® *GFP, TGFBR3* and *PLAU* esiRNA (Sigma). *PTGS2* knockdown was performed using control and *PTGS2* DsiRNA (Integrated DNA Technologies). MTT assays were performed using Thiazolyl Blue Tetrazolium Bromide (Sigma).

### Transcriptome sequencing and mapping

Mouse fur was removed with a trimmer and skin was rinsed with ethanol 75% (Sigma) followed by DEPC water (Sigma). Whole skin punch biopsies (100 mg) were minced in 1 ml of ice cold TRI-reagent (Sigma) using the Ultra-Turrax® T10 homogenizer (IKA), and total RNA was extracted following the manufacturer’s instructions. RNA was analyzed using a Bioanalyser (Agilent Technology Inc). RNA integrity numbers for the analysed samples ranged from 9 to 12. mRNA libraries were prepared following the standard Illumina protocol. They were sequenced on the Illumina Nextseq platform at the Ramaciotti Centre for Genomics UNSW, to produce approximately 30 million, 75 nucleotide paired-end reads per sample (Reads 1 and 2). Reads were mapped to the Ensembl *Mus musculus* genome (GRCm38) provided by Illumina iGenomes (cufflinks.cbcb.umd.edu/igenomes.html). Mapping was performed with Tophat2 (v 2.0.8)^47^ calling Bowtie2 (v 2.1.0)^48^ using the default settings. HTSeq-count (Python package HTSeq, python v 2.7.3)^49^ was used to generate counts of reads uniquely mapped to annotated genes using the GRCm38 annotation gtf file, 26579 genomic features had at least one read assigned. Raw sequencing data are available at the Gene Expression Omnibus, GSE 121194.

### Differential gene expression analysis

Differential expression analysis was performed using edgeR (v 3.8.6)^9^ and voom (limma)^10^. Tables of raw counts generated using HTSeq-count were used as input in both analyses. Low count transcripts were excluded and only those genes with at least 1 count per million (cpm) in at least 3 samples were used for analysis. A normalization factor was calculated using the trimmed mean of M values method^50^. The functions estimateGLMCommonDisp and estimateGLMTagwiseDisp were used to estimate dispersion. Following this we tested for differential expression using the generalized linear model function glmFit and glmLRT. The DGEList object created in edgeR was used in the voom analysis. The voom function was used to transform the count data prior to applying the lmFit and eBayes functions to test for differential expression. The Benjamini-Hochberg correction was used with a false discovery cut-off of 0.05 in all methods.

### Functional analysis

GOrilla^51^ was used to investigate the functional associations of the differentially expressed genes. The genes tested for differential expression, after exclusion of lowly expressed genes were used as background (*n* = 13768) in the gene ontology analysis. The Gene Ontology (GO) terms in the category Biological Process were tested for overrepresentation using the hypergeometric test and *P* values were corrected using the Benjamini & Hochberg FDR correction. We used the REVIGO tool^52^ to reduce redundancy and produce a more compact list of gene ontology terms associated with the differentially expressed genes. The list of genes found to contain the M6 motif were tested for gene ontology enrichment using the DAVID tool ^53^.

### Motif analysis

We firstly used Ensembl biomart to obtain the 3′ UTR for the transcripts associated with the 260 differentially expressed genes found using voom. This produced 526 sequences. This set of sequences was then interrogated for the presence of each of the 20 binding motifs identified by Zanconato et al. using the FIMO (Find Individual Motif Occurrences) algorithm^17^ of the MEME Suite of programs. FIMO returned a *P* value and mutlitest adjusted q value for the presence of each of the tested motifs in the 526 sequences interrogated. Only those occurrences which had a *P* value < 1 E0-4 and a *q* value < 0.2 were retained.

### Tissue processing and histological and immunofluorescence staining

Full thickness mice skin tissues were fixed in paraformaldehyde 4%, paraffin embedded, sectioned, and histology-stained following routine procedures. Antigen retrieval was performed using 10 mM sodium citrate buffer (pH 6.0) and a Milestone RHS-1 Microwave at 110 °C for 5 min. Tissue sections were immunostained using previously standardized methods, and confocal images were captured using an Olympus FV1200 laser scanning confocal microscope. Immuno-signal intensity was quantified in a semi-automated fashion using ImageJ software. Primary and secondary antibody information is displayed in Supplementary Table S5.

### Western blotting and qRT-PCR analysis

qRT-PCR was performed on mice from the same cohort as used for RNA-Seq analysis. Whole skin punch biopsies (100 mg) were minced in 1 ml of ice cold TRI-reagent (Sigma) using the Ultra-Turrax® T10 homogenizer (IKA). Total RNA and protein from mouse skin biopsy and HaCaT keratinocytes were extracted following the manufacturer’s instructions. Protein lysates were analyzed by western blot, intensity of bands was quantified with ImageJ software and normalized to GAPDH, GSK3β or β-catenin. Primary and secondary antibody information is displayed in Supplementary Table S5. Quantitative RT–PCR assays were carried out using Fast SYBR® Green Master Mix (Thermo Fisher Scientific, 4385612) and Mx3000P qPCR System (Agilent Technologies), and were analysed by the comparative cycle time method, normalizing to *18* *S* ribosomal or *GAPDH* RNA levels. Human and mouse primer information is available in Supplementary Table S6.

### Statistical analysis

Statistical significance was determined by Student’s unpaired *t*-tests. Error bars represent mean ± SEM. Asterisks indicate statistical significance, where *P* < 0.05 was used as significance cut-off.

## Electronic supplementary material

Supplemental Table 1

Supplemental Table 2

Supplemental Table 3

Supplemental Table 4

Supplemental Table 5

Supplemental Table 6

Supplemental Table 7

Supplementary figure legends
