# Supplementary material for: Plau and Tgfbr3 are YAP-regulated genes that promote keratinocyte proliferation
Source: Cell Death Dis. 2018 Oct 31;9(11):1106. doi: 10.1038/s41419-018-1141-5 (PMC6208416; doi:10.1038/s41419-018-1141-5)
Supplement: Supplementary file 8 — Supplementary figure legends [file 41419_2018_1141_MOESM8_ESM.docx]

**Supplementary Tables**

**Supplementary Table S1**: **Differentially expressed genes found by edgeR and voom.** This file contains the analysis results for all genes tested using the Bioconductor packages edgeR and limma (voom).

**Supplementary Table S2: Genes found to be differentially expressed in RNA-Seq tested by RT-qPCR.**

**Supplementary Table S3: YAP/TAZ/TEAD motifs found in 3’ UTR of differentially expressed genes.** This file sets out the genes found to harbor one or more of the YAP/TAZ/TEAD motifs identified by Zanconato et al. 2015.

**Supplementary Table S4: RNA-Seq results for 17 genes the subject of experimental validation.**

**Supplementary Table S5: Primary and Secondary antibodies used in Western blots.**

**Supplementary Table S6: Human and Mouse primers used in qPCR.**

**Supplementary Table S7: Comparison of the outcomes of this and the Walko et al study** (21)**.**
